# Seasonal Variation in Zooplankton Community Structure and Its Environmental Drivers in the Coastal Waters of Lanshan Port

**DOI:** 10.3390/biology15090679

**Published:** 2026-04-25

**Authors:** Liang Zhang, Lan Wang, Cong Fang, Yinglu Ji, Sichao Pu, Huihui Tao, Haizhou Zhang, Yumeng Liu

**Affiliations:** 1 North China Sea Marine Forecasting and Hazard Mitigation Center, Ministry of Natural Resources, Qingdao 266100, China; zhangliang@ncs.mnr.gov.cn (L.Z.); wanglan_bhybzx@ncs.mnr.gov.cn (L.W.); jiyinglu@ncs.mnr.gov.cn (Y.J.); pusichao@ncs.mnr.gov.cn (S.P.); taohuihui@ncs.mnr.gov.cn (H.T.); zhanghaizhou@ncs.mnr.gov.cn (H.Z.); 2 Zhejiang Environmental Technology Co., Ltd., Hangzhou 310030, China; fczjshjkj@163.com

**Keywords:** zooplankton community, spatio-temporal distribution, environmental drivers, dominance, diversity

## Abstract

Coastal port ecosystems serve as critical interfaces between marine environments and anthropogenic activities, yet zooplankton community dynamics in these transitional zones remain poorly understood. This study investigated seasonal variations in zooplankton assemblages and their environmental drivers in the coastal waters surrounding Lanshan Port, northern Yellow Sea, through quarterly field surveys spanning spring to winter. A total of 33 zooplankton species and 16 planktonic larval categories were identified, with Hydromedusa, Copepoda, and planktonic larvae comprising the three dominant groups. Marked seasonal disparities were observed in species richness (spring: 21 species and 11 larvae categories; winter: 8 species and 3 larvae categories), biomass (autumn: 333.7 mg/m^3^; winter: 34.0 mg/m^3^), and abundance (spring: 185.3 ind/m^3^; winter: 25.7 ind/m^3^). Notably, *Aidanosagitta crassa* maintained perennial dominance across all seasons. Principal component analysis of dominant zooplankton taxa across seasons indicated that the first two principal components explained 70.05% and 15.97% of the total variance in zooplankton community structure, respectively, with distinct seasonal clustering of sampling sites along PC1 reflecting pronounced seasonal succession in community composition. Redundancy analysis revealed seasonal-specific correlations between dominant taxa and nutrients: nitrate concentration was negatively correlated with the relative abundance of Sergestidae in both spring and summer, whereas ammonium concentration was negatively correlated with Hydromedusa; by contrast, the abundances of Chaetognatha and Tunicata exhibited a significant positive correlation with nitrate. We also found water temperature only drove communities in autumn, while salinity had little effect. These findings elucidate the mechanisms structuring zooplankton communities in temperate coastal port ecosystems and underscore the necessity of seasonally resolved monitoring frameworks for effective marine environmental management.

## Simple Summary

Zooplankton occupy a critical ecological niche in marine ecosystems, acting as a key trophic link between primary producers and higher trophic levels. However, their dynamics in port environments remain poorly understood. This study focuses on the zooplankton community in the coastal waters of Lanshan Port, Yellow Sea. Based on four quarterly surveys, a total of 33 zooplankton species and 16 larval taxa were identified. Obvious seasonal variations were observed in both species richness and abundance, with the highest values occurring in spring. Copepods, Hydromedusa, and planktonic larvae represented the dominant taxonomic groups. The principal component analysis and redundancy analysis demonstrated that season and nutrient concentrations exerted dominant controls over the composition of dominant zooplankton species. This research establishes a critical ecological baseline for the Lanshan Port area, emphasizing the necessity of long-term seasonal monitoring and integrated management strategies to conserve marine biodiversity and support sustainable development in coastal port ecosystems.

## 1. Introduction

As critical secondary producers in marine ecosystems, zooplankton serve as irreplaceable hubs connecting primary producers to higher trophic levels, while profoundly participating in key biogeochemical processes such as the marine carbon cycle [1,2]. Characterized by small body size, high metabolic rates, and planktonic lifestyles, they can rapidly respond to subtle changes in the marine environment [3,4], rendering them ideal bio-indicators for assessing coastal ecological quality and environmental disturbances [5]. Spatio-temporal heterogeneity of zooplankton communities in coastal waters worldwide is typically driven by natural factors, including hydrodynamics, temperature, and salinity [6,7,8]; for instance, species richness, biomass, and abundance are generally significantly higher in warmer seasons than colder seasons [9,10]. Among various taxa, copepods and chaetognaths are widely distributed with stable populations, often dominating coastal zooplankton communities, and changes in their community structure can directly reflect the health status of ecosystems [11,12,13].

With the continuous expansion of global port construction, human activities such as reclamation [14], sediment dredging [15], and ship navigation [16] have become major disturbance sources to zooplankton habitats in coastal areas [17]. Reclamation projects directly alter coastal topography and hydrological connectivity, potentially leading to the replacement of dominant zooplankton taxa and significant declines in species richness and abundance [18,19,20], although some indicators may show partial natural recovery following project cessation [21]. Intensive ship activities and pollutant discharge in port areas further exacerbate community disturbances [22,23]; for example, the dispersed crude oil alters the growth and survival of copepods [23,24], and the zooplankton in ships’ ballast water poses a risk of biological invasion [25,26]. Additionally, changes in environmental factors induced by port construction, such as increased suspended solids and abnormal turbidity [27], indirectly affect zooplankton communities by influencing phytoplankton productivity [28], ultimately forming ecological patterns distinct from natural waters.

Rizhao Lanshan Port, an important comprehensive port in northern China, features the dual characteristics of busy shipping, frequent engineering activities, and significant ecological functions [29,30]. With the development and upgrading of Lanshan Port [31], this area has undergone long-term reclamation. Previous studies have focused on the impact of reclamation projects at Lanshan Port on zooplankton communities in adjacent waters. For instance, land reclamation exerted a certain impact on the number of species and abundance of zooplankton [32]. Specifically, the reclamation projects induced changes in the dominant zooplankton groups in nearby waters, along with a decrease in species number and abundance; after the reclamation ceased, the dominant groups, species number, and abundance all showed a certain degree of recovery [33]. In addition, the community structures of zooplankton in the waters adjacent to the North Operation Area of Rizhao Lanshan Port in spring and autumn 2015 were investigated [34]. However, these previous studies mainly focused on zooplankton surveys in the sea area during autumn, lacking both multi-seasonal result comparisons and attention to the influence of water quality and other environmental factors on zooplankton.

Based on this, the present study conducted multi-seasonal and multi-station field investigations from 2022 to 2024 to systematically analyze the species composition, dominant species, abundance, and biomass of zooplankton in the waters adjacent to Lanshan Port, as well as the effects of environmental factors on their community structure. This study aims to clarify the spatio-temporal distribution patterns of the zooplankton community and shed light on the effects of seasons and water quality on its structure and dynamics.

## 2. Materials and Methods

### 2.1. Zooplankton Sampling and Environmental Parameter Measurement

**Figure 1:**
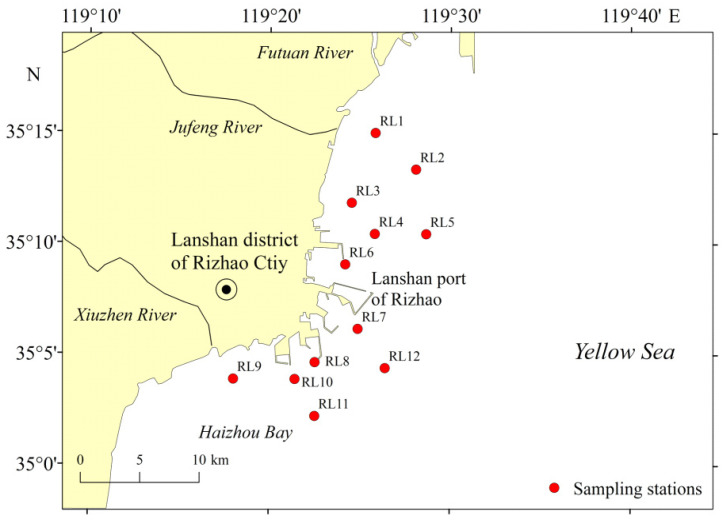
Sampling stations (RL1 to RL12) of the zooplankton in the coastal waters adjacent to Lanshan Port.

A total of 12 sampling stations were established in the coastal waters adjacent to Lanshan Port (Figure 1). Four seasonal cruises were carried out: November 2022 (autumn), June 2023 (summer), December 2023 (winter), and May 2024 (spring). Water depths at the sampling stations ranged from 2.9 m at station RL9 to 21.5 m at station RL7 (Table S1). Zooplankton samples were collected using a Type I shallow-water plankton net (mouth area: 0.20 m^2^, mesh size: 505 μm) (Qingdao Haohai Instrument Co., Ltd., Qingdao, China) by vertical towing following the specifications of GB17378.7-2007 [35]. All samples were immediately preserved in 5% buffered formalin solution(Yantai Yuandong Fine Chemical Co., Ltd., Yantai, China) upon collection. Zooplankton species identification and enumeration were performed based on morphological characteristics under a stereomicroscope (ZEISS Stemi 508, Oberkochen, Germany) [36,37]. Wet weight was measured [35] using an electronic balance (Sartorius BS210S, Goettingen, Germany) with a precision of 0.0001 g.

Surface water temperature was measured in situ using a digital water temperature meter (HD23-WTR-2, Beijing Haifuada Technology Co., Ltd., Beijing, China). During zooplankton sampling, water samples were simultaneously collected for the analysis of salinity, nutrients, and chlorophyll *a*. Water salinity was determined using a SYA2-2 laboratory salinometer (Tianjin Haiwei Technology Development Co., Ltd., Tianjin, China). Nutrient concentrations were measured with an ultraviolet-visible spectrophotometer (TU1810, Beijing Puxi General Instrument Co., Ltd., Beijing, China) following the specifications of GB 17378.4-2007 [38]. Briefly, ammonia nitrogen was determined by the bromate oxidation method; nitrite nitrogen by the N-(1-naphthyl) ethylenediamine spectrophotometric method; nitrate nitrogen by the zinc-cadmium reduction method; and reactive phosphate by the phosphomolybdenum blue spectrophotometric method. Chlorophyll *a* was analyzed according to GB 17378.7-2007 [35] using a visible-light spectrophotometer (722S, Shanghai Yitian Precision Instrument Co., Ltd., Shanghai, China) with the standard spectrophotometric method.

### 2.2. Data Statistics and Analysis

#### 2.2.1. Abundance, Wet Weight Biomass, and Dominance of Zooplankton

The abundance (*A*) [39], wet weight biomass (*B*) [39], and dominance (*Y*) [40] of the captured zooplankton were calculated based on the following formulae.

(1) $$A=\frac{N}{V}$$

(2) $$B=\frac{W}{V}$$

(3) $$Y=\frac{n_{i}}{N}\times f_{i}$$

where *N* was the total number of zooplankton collected at each station (ind.); *V* was the volume of filtered seawater per station (m^3^); *W* was the zooplankton sample wet weight (mg); *n_i_* was the individual number of the *i*-th zooplankton species (ind); *N* was the total number of zooplankton (ind.); *f_i_* was the frequency of occurrence of the *i*-th zooplankton taxon across all sampling stations. Species with *Y* ≥ 0.02 were defined as dominant species [41].

#### 2.2.2. Biodiversity Patterns of the Zooplankton Communities

Biodiversity patterns of the zooplankton communities were evaluated using the traditional Shannon–Wiener diversity index (*H′*) [42], Margalef taxonomic richness index (*d*) [43], and Pielou evenness index (*J*) [44]. These indices were calculated based on the following formulae.

(4) $$H^{\prime }=\sum_{i=1}^{s}P_{i}{\log_{2}P}_{i}$$

(5) $$d=\frac{S-1}{\log_{2}N}$$

(6) $$J=\frac{H^{\prime }}{H_{max}}$$

where *S* was the total number of taxa in a sample; *P_i_* was the ratio of the individual number of the *i*-th zooplankton taxon to the total number of zooplankton individuals (*N*); *H_max_* was log_2_S.

#### 2.2.3. Statistics and Correlation Analysis

IBM SPSS Statistics 27.0 (IBM Corporation, Armonk, NY, USA) was used to conduct one-way ANOVA and Tukey’s HSD post hoc test for pairwise comparison of the zooplankton abundance, biomass, and diversity indices, environmental factors, as well as diversity and evenness indices of the dominant species across seasons, with significance thresholds set at *p* < 0.05 (significant) and *p* < 0.01 (highly significant). Principal component analysis (PCA) was employed to examine seasonal variations in community structure using a matrix constructed based on the relative abundance of dominant zooplankton groups at each sampling site. All analyses were conducted in R version 4.5.1. Redundancy analysis (RDA) was performed using Origin 2024 (OriginLab Corporation, Northampton, MA, USA) to examine the relationships between zooplankton relative abundance and environmental factors across seasons. Prior to analysis, all biotic and abiotic data (x) were ln(x + 1) transformed to down-weight the influence of dominant taxa. The Permutation test was conducted to evaluate the significance of the explanatory variables, and a forward selection procedure was applied to remove the redundant data. Multicollinearity among environmental variables was assessed using variance inflation factors (VIF). Variables with a correlation coefficient |r| > 0.8 and VIF > 20 were excluded from the final model. The significance of the overall model and individual environmental variables was evaluated using permutation tests (999 permutations), with significance set at *p* < 0.05.

## 3. Results

### 3.1. Environmental Parameters of the Coastal Waters Adjacent to Lanshan Port

Surface water temperature in the coastal waters of Lanshan Port varied significantly among seasons (*p* < 0.05), showing a clear seasonal hierarchy: highest in summer (22.34 °C), followed by spring (20.10 °C) and autumn (14.21 °C), and lowest in winter (5.49 °C). During spring, autumn, and winter, the overall water temperature was slightly higher at southern stations than at northern stations (Table 1 and Table S2). In contrast, no significant spatial differences in temperature were observed in summer. Water salinity also showed obvious seasonal variation, with the highest mean value in winter (29.733) and the lowest in autumn (29.081); these values differed significantly from those observed in the other two seasons (*p* < 0.05). Spatially, salinity displayed a consistent increasing gradient from nearshore to offshore waters. The concentrations of nitrite, nitrate, and ammonium were all the lowest in summer, with mean values of 4.57, 66.21, 9.86, and 80.64 μg/L, respectively. Nitrate and ammonium peaked in autumn at 206.17 and 26.63 μg/L, respectively, whereas nitrite showed similar values in autumn (25.04 μg/L) and winter (25.39 μg/L). Mean phosphorus concentration was the lowest in spring (1.76 μg/L), significantly lower than in other seasons (*p* < 0.05), with values at stations RL1, RL2, and RL5 below 1 μg/L. In comparison, phosphorus peaked in autumn at 8.58 μg/L, with no significant difference from summer. Two extremely high phosphorus values (>20 μg/L) were recorded at stations RL1 and RL9 in autumn, while only station RL12 in winter showed a relatively high level (10.1 μg/L) during other seasons. Mean chlorophyll *a* concentrations were higher in summer (3.74 μg/L) and winter (3.18 μg/L) but lower in spring (1.77 μg/L) and autumn (1.26 μg/L), with significant seasonal differences (*p* < 0.05). In particular, chlorophyll *a* concentrations at stations RL1, RL6, and RL9 exceeded 5 μg/L in summer, whereas all stations showed values below 2 μg/L in autumn.

### 3.2. Zooplankton Species Composition

A total of 33 zooplankton species and 16 planktonic larval taxa were identified in the coastal waters adjacent to Lanshan Port during the present study (Table 2 and Table S3). Excluding planktonic larvae, Copepoda displayed the highest species richness (12 species), accounting for 24% of the total zooplankton species, followed by Hydromedusae (10 species). All other zooplankton taxa, including Siphonophora, Ctenophora, Amphipoda, Sergestidae, Chaetognatha, and Tunicata, were represented by no more than three species. Specifically, Ctenophora and Chaetognatha were each represented by only one species (2% of the total species pool), while Amphipoda comprised three species. Regarding seasonal variations, the highest species richness occurred in spring, with 21 zooplankton species and 11 larval taxa identified, followed by summer (19 zooplankton species and 12 larval taxa) and autumn (19 zooplankton species and 6 larval taxa). The lowest species richness was recorded in winter (8 zooplankton species and 3 larval taxa). Hydromedusa, Copepoda, and planktonic larvae were the three dominant groups across all seasons, contributing 72.0% (autumn) to 84.4% (spring) of the total species number. Despite their overall dominance, the relative species proportion of each zooplankton group varied markedly among seasons. For instance, planktonic larvae dominated in spring and summer, whereas Copepoda accounted for a higher proportion in autumn and winter.

### 3.3. Zooplankton Biomass and Abundance

**Figure 2:**
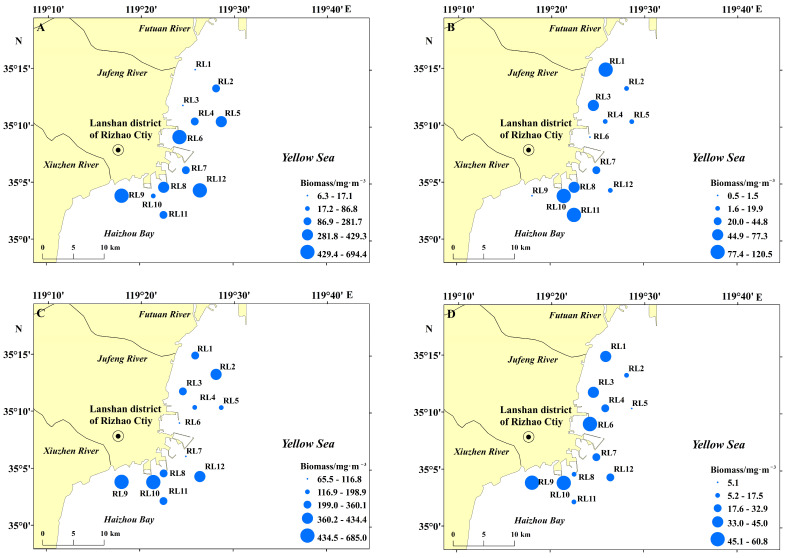
Distributions of zooplankton wet weight biomass in the sea area surrounding Lanshan Port across seasons. (**A**) Spring; (**B**) Summer; (**C**) Autumn; (**D**) Winter.

Zooplankton wet weight biomass showed significant seasonal (*p* < 0.05) and spatial variability in the study area (Figure 2). In spring, mean zooplankton biomass was 306.8 ± 226.8 mg/m^3^, ranging from 6.3 to 694.4 mg/m^3^, with the minimum at station RL3 and the maximum at station RL6. In summer, biomass decreased sharply to 50.3 ± 45.7 mg/m^3^, varying between 0.5 and 120.5 mg/m^3^, with the lowest value at station RL6 and the highest at station RL11. Mean biomass peaked in autumn at 333.7 ± 180.6 mg/m^3^, ranging from 65.5 to 685.0 mg/m^3^, with the minimum at station RL7 and the maximum at station RL9. Winter exhibited the lowest mean biomass (34.0 ± 18.3 mg/m^3^) across all seasons, with a narrow range of 5.1 to 60.8 mg/m^3^; the minimum was recorded at station RL5 and the maximum at station RL6. Spatially, zooplankton biomass displayed heterogeneous distribution patterns across seasons. In spring, biomass generally showed a north-to-south gradient, with lower values in the northern region and higher values in the southern area. Summer and autumn showed similar spatial patterns: biomass was relatively lower in the central zone near Lanshan Port but high in both the northern and southern regions. In winter, a clear nearshore-offshore gradient was observed, with biomass decreasing gradually from inshore to offshore waters.

**Figure 3:**
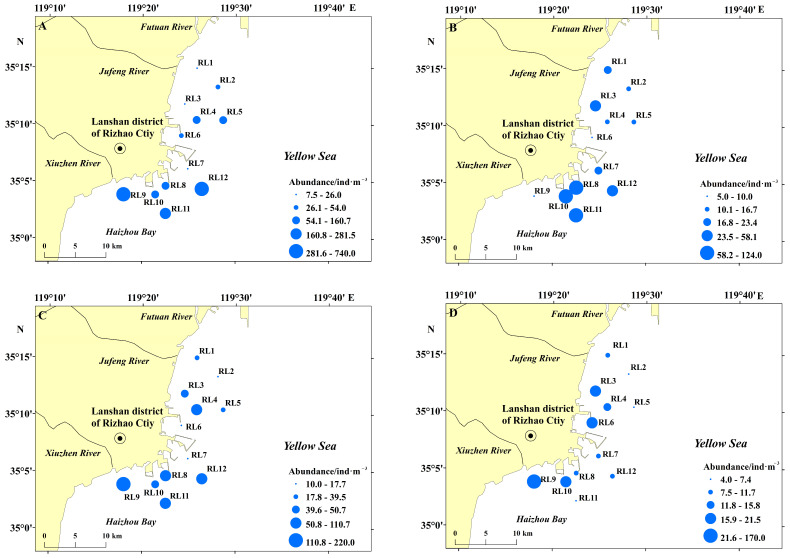
Distribution of zooplankton abundance in the sea area surrounding Lanshan Port across seasons. (**A**) Spring; (**B**) Summer; (**C**) Autumn; (**D**) Winter.

The spatial variation in zooplankton abundance was similar to that of wet weight biomass (Figure 3). Overall, zooplankton abundance exhibited south-to-north and nearshore-offshore gradients, decreasing gradually from southern to northern regions and from inshore to offshore waters. Significant seasonal differences in zooplankton abundance were detected (*p* < 0.05), with a decreasing order of mean abundance: spring (185.3 ind/m^3^) > autumn (63.9 ind/m^3^) > summer (39.7 ind/m^3^) > winter (25.7 ind/m^3^). The maximum total abundance in spring, autumn, and winter was recorded at station RL9, with values of 740.0 ind/m^3^, 220.0 ind/m^3^, and 170.0 ind/m^3^, respectively. By contrast, the highest abundance in summer occurred at station RL11 (124.0 ind/m^3^). Interestingly, the station with the minimum abundance varied seasonally: station RL3 in spring, station RL6 in summer, station RL7 in autumn, and station RL5 in winter.

### 3.4. Dominance of Zooplankton

A total of 8 dominant zooplankton species (adults) and 7 planktonic larval taxa were identified (Table 3). The adult dominant species included 1 hydromedusa, 2 siphonophores, 2 copepods, 1 amphipod, 1 chaetognath, and 1 tunicate. Seasonal variations in the dominant assemblages were pronounced, with *Aidanosagitta crassa* being a consistent dominant species across all seasons. In spring, the dominant community consisted of 4 adult zooplankton species and 4 larval taxa. Fish eggs were the most dominant taxon (*Y* = 0.25), while *Calanus sinicus* had the highest abundance (89.4 ind.∙m^−3^). In summer, the dominant groups included 2 adult species and 5 larval taxa. Porcellana Zoea larvae were the most dominant taxon (*Y* = 0.16) and had the highest abundance (12.2 ind.∙m^−3^). In contrast, a total of 5 adult species and 2 larval categories constituted the dominant community in autumn; *A. crassa* was the most dominant (*Y* = 0.32) and abundant (*A* = 23.3 ind.∙m^−3^) species. In winter, only 2 adult species were identified as dominant taxa: *A. crassa* was the primary dominant species (*Y* = 0.71, *A* = 10.2 ind.∙m^−3^), followed by *C. sinicus* as the second dominant species.

### 3.5. Zooplankton Community Diversity

**Figure 4:**
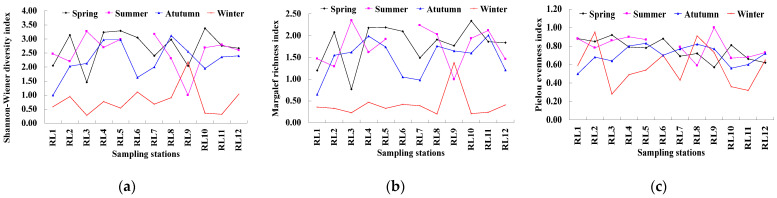
Shannon-Wiener diversity index (**a**), Margalef richness index (**b**), and Pielou evenness index (**c**) of zooplankton in the sea area surrounding Lanshan Port across seasons. No values for the station RL6 in summer because only one species was identified.

The Shannon-Wiener diversity index of the zooplankton community in the coastal waters adjacent to Lanshan Port showed seasonal differences, with the highest mean value of 2.71 in spring and the lowest mean value of 0.81 in winter (Figure 4). The Shannon-Wiener diversity indices of the sampling stations in spring, summer, autumn and winter were 1.46 to 3.38, 1.00 to 3.27, 1.00 to 3.12 and 0.28 to 2.18, respectively, and that in winter was significantly lower than other seasons (*p* < 0.05). The highest Shannon-Wiener diversity index in each season was recorded at station RL10 in spring (3.38), RL3 in summer (3.27), RL8 in autumn (3.12) and RL9 in winter (2.18). In contrast, the lowest values of this index were observed at station RL3 in spring (1.46) and winter (0.28), RL9 in summer (1.00) and RL1 in autumn (1.00). The Margalef richness index also exhibited seasonal differences, but only in winter was the value significantly different from other seasons (*p* < 0.05), with the highest mean value of 1.81 in spring and the lowest mean value of only 0.41 in winter. Specifically, the maximum value of the Margalef richness index (2.35) was detected at station RL3 in summer, while the minimum value (0.20) was found at station RL8 in winter. Significant seasonal differences in the Pielou evenness index were detected between spring and winter, as well as between summer and winter (*p* < 0.05), with mean values of 0.76, 0.80, 0.70 and 0.58 in spring, summer, autumn and winter, respectively. The highest evenness index (1.00) was measured at station RL9 in summer, whereas the lowest value (0.28) was documented at station RL3 in winter.

### 3.6. Relationships Between Dominant Zooplankton Taxa and Environmental Factors

**Figure 5:**
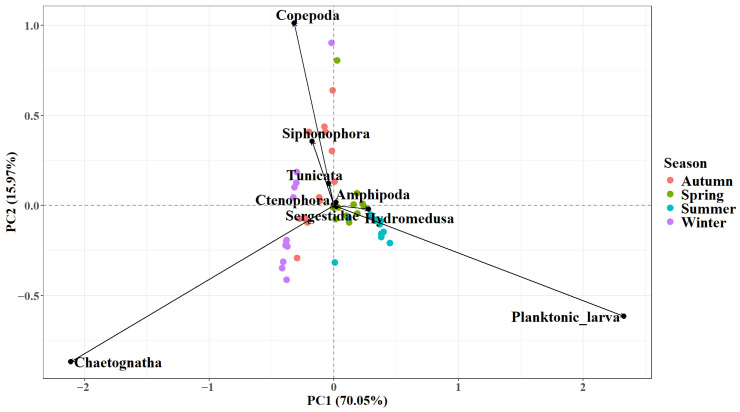
The principal component analysis of dominant zooplankton taxa across seasons.

The principal component analysis of dominant zooplankton taxa across seasons showed that the first two principal components explained 70.05% and 15.97% of the total variance in zooplankton community structure, respectively (Figure 5). Distinct seasonal clustering of sampling sites was observed along PC1, indicating pronounced seasonal succession in community composition. Amphipoda and planktonic larvae loaded positively on PC1, which was associated with summer samples, whereas Chaetognatha, Siphonophora, and Copepoda showed negative loadings, corresponding to winter conditions. Planktonic larvae exhibited the longest vector, followed by Chaetognatha, indicating their strong influence on the observed seasonal gradient. Narrow angles among Copepoda, Siphonophora, and Tunicata suggested co-occurrence, whereas Chaetognatha was nearly orthogonal, indicating independent distribution patterns.

**Figure 6:**
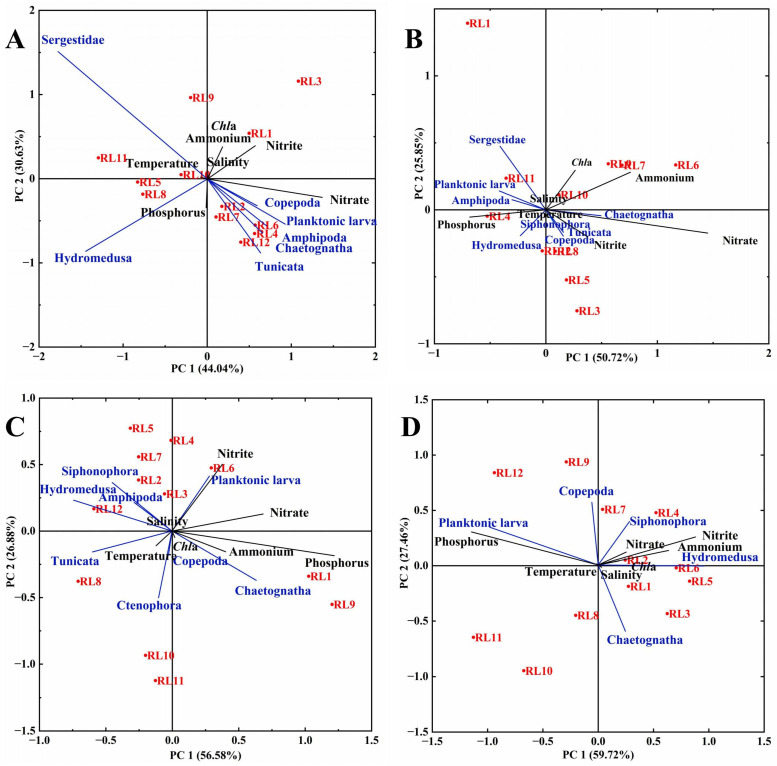
The RDA between the relative abundance of zooplankton groups and environmental factors in the sea area surrounding Lanshan Port across seasons. (**A**) Spring; (**B**) Summer; (**C**) Autumn; (**D**) Winter.

Redundancy analysis of dominant zooplankton abundance and environmental variables across seasons revealed that in spring, the relative abundance of Sergestidae showed a significant negative correlation with nitrate concentration (*p* < 0.05), whereas Ctenophora, planktonic larvae, Amphipoda, Chaetognatha, and Tunicata exhibited positive correlations with nitrate (Figure 6). Additionally, Hydromedusa abundance was significantly negatively correlated with both nitrite and ammonium concentrations (*p* < 0.05). In summer, the relative abundance of Sergestidae, planktonic larvae, and Amphipoda displayed significant negative correlations with nitrate and nitrite, while Chaetognatha, Tunicata, Siphonophora, and Copepoda showed positive correlations with the two nutrients (*p* < 0.05). In addition, Hydromedusa abundance was significantly negatively correlated with ammonium (*p* < 0.05). In autumn, Tunicata abundance was significantly and negatively correlated with nitrate (*p* < 0.01), whereas planktonic larvae were significantly and positively correlated with nitrite (*p* < 0.01). Furthermore, Siphonophora, Hydromedusa, and Amphipoda were significantly negatively correlated with phosphorus and ammonium (*p* < 0.05), while Chaetognatha showed significant positive correlations with these variables (*p* < 0.05). In winter, planktonic larvae abundance was significantly positively correlated with phosphorus (*p* < 0.05), while Hydromedusa abundance showed a significant negative correlation with phosphorus (*p* < 0.05). Both Siphonophora and Hydromedusa were positively correlated with nitrite, nitrate, and ammonium concentrations.

## 4. Discussion

### 4.1. Seasonal Patterns of Zooplankton Community Structure

The present study revealed pronounced seasonal variations in zooplankton community structure in the coastal waters adjacent to Lanshan Port, with species richness peaking in spring (21 species and 11 larval categories) and declining to a minimum in winter (8 species and 3 larval categories). This seasonal pattern aligns with observations from other temperate coastal ecosystems, where temperature and food availability are primary drivers of zooplankton succession [45]. Despite potential influences from inter-annual variability and month-specific fluctuations [10], which preclude generalization of seasonal patterns, our results nevertheless reveal distinct differences across seasonally sampled stations. The dominance of copepods and hydromedusae in Lanshan Port waters is consistent with the community composition reported before in the sea area near Lanshan Port, where arthropods, planktonic larvae, and cnidarians constituted the principal components of the zooplankton assemblage [32,33,34,46]. These results suggested a relatively stable zooplankton community composition in this area. Globally, warming has been associated with increased proportions of jellyfish (including hydromedusae) in the zooplankton composition in some sea areas [47], which may partly explain the relatively high hydromedusa diversity observed in summer in this study. Furthermore, both top-down and bottom-up controls contribute to the higher number of hydromedusa species [48,49], and this also results in the negative correlation between chlorophyll *a* and Hydromedusa as shown in the RDA result. Notably, the seasonal alternation between planktonic larvae dominance in spring-summer and copepod dominance in autumn-winter suggests a shift in community control mechanisms driven by possibly physical forcing [50].

The identification of *A. crassa* as the perennial dominant species, particularly its overwhelming dominance in winter (*Y* = 0.71), indicates its remarkable adaptability to low-temperature and oligotrophic conditions. This finding corroborates previous studies identifying *A. crassa* as the most dominant chaetognath species in the shallow waters of northern Liaodong Bay and the Yellow Sea [45]. Similarly, *C. sinicus*, a key copepod species in the Yellow Sea ecosystem, exhibited seasonal succession from spring dominance to winter sub-dominance, reflecting its life history strategy of diapause during unfavorable conditions [51].

The significant decline in zooplankton diversity indices during winter (Shannon-Wiener index: 0.81 ± 0.65) and the concurrent reduction in species richness to only 8 species and 3 larval categories indicate substantial seasonal bottleneck effects on community stability. This winter reduction in functional diversity may compromise the resilience of the coastal ecosystem to environmental perturbations, as low-diversity communities typically exhibit diminished functional redundancy [52]. The pronounced spatial heterogeneity observed in spring, with station RL3 exhibiting both the highest nitrogen concentrations and lowest zooplankton biomass, suggests localized eutrophication impacts that warrant targeted monitoring.

### 4.2. Environmental Drivers of Dominant Zooplankton Abundance

The RDA and PCA results demonstrated seasonal distinct relationships between dominant zooplankton communities and environmental variables, accompanied by marked seasonal succession in community composition, emphasizing the complex influence of physical forcing on zooplankton community structure. In spring, the nitrogenous nutrients had a significant effect on the dominant zooplankton community, especially the negative correlation between Sergestidae and nitrate, as well as between Hydromedusa and nitrite. This result supported the preference of both taxa for low-nutrient waters [53,54]. This finding is particularly relevant given the low nitrogen concentrations observed in spring and summer, accompanied by a high abundance of the representative species *Clytia hemisphaerica*. Planktonic larvae showed positive correlations with all phosphorus and nitrogenous nutrients in autumn and winter. This pattern is typical in coastal areas, where enhanced vertical mixing during autumn and winter elevates nutrient concentrations via upward transport of nutrient-rich bottom water [55]. Meanwhile, most benthic taxa release larvae during these seasons. The synchrony between nutrient elevation and larval release results in positive correlations, reflecting coupling between hydrodynamic processes and benthic reproductive rhythms, rather than a direct promoting effect of nutrients on meroplankton [54].

Water temperature is one of the most important environmental factors affecting zooplankton community structure [9]. The RDA results in the present study showed that water temperature had weak explanatory power for the dominant zooplankton groups in spring, summer and winter, and only became a key driving factor in autumn, which may be related to the strongest spatial temperature gradient occurring in autumn (Table S2). However, the contrasting patterns of biomass distribution—with spring and autumn peaks versus summer and winter minima—suggest that temperature alone does not govern zooplankton production. Instead, there is a mismatch between biomass and abundance in spring and autumn (lower biomass but higher abundance in spring). Although size-fractionated data are not available to confirm this interpretation, we speculate that it indicates a community shift toward smaller-bodied species, a common response to warm-water stratification and reduced nutrient mixing [50]. Although Moya-Lucena & Duggan suggested salinity was the main driver of zooplankton composition and diversity [56], salinity showed little influence on the relative abundance of dominant zooplankton groups in all seasons in this study, likely due to the slight annual variations in salinity observed in this region.

As a proxy for phytoplankton biomass, chlorophyll *a* concentrations were relatively high in summer and winter, but zooplankton abundance and biomass remained low, showing a negative correlation. In general, appropriate phytoplankton density can promote the growth and reproduction of zooplankton [57]. However, phytoplankton in summer are mostly large, inedible, and toxic species, resulting in the failure of bottom-up control [58]. In winter, low temperatures inhibit the metabolism and reproduction of zooplankton, which are dominated by dormant, low-metabolic, and small-sized individuals [59]. Similarly, in spring and autumn, bottom-up regulation of phytoplankton on zooplankton exceeds top-down control of zooplankton on phytoplankton [60], leading to higher zooplankton biomass and abundance.

## 5. Conclusions

This study provides the first multi-seasonal, systematic survey of zooplankton communities with concurrent environmental monitoring assessment in the coastal waters adjacent to Lanshan Port, revealing significant seasonal and spatial variations in species composition, biomass, and diversity. The zooplankton community is dominated by copepods and hydromedusae, with *Aidanosagitta crassa* serving as a key indicator species across seasons. Environmental factors, particularly water temperature and nutrient concentrations, exhibit distinct seasonal influences on dominant zooplankton distribution patterns. The observed seasonal succession—from high-diversity, high-abundance communities in spring to low-diversity, low-abundance in winter—reflects the dynamic response of zooplankton to physical forcing and trophic interactions in this temperate coastal system. These findings establish a baseline for assessing the ecological impacts of port expansion on zooplankton communities in the Yellow Sea coastal zone, emphasizing the need for integrated long-term monitoring programs that track both biological and physicochemical parameters to ensure sustainable management of coastal marine resources.

## Figures and Tables

**Table 1:** Mean environmental factor values in the coastal waters surrounding Lanshan Port (Mean value ± S.D.).

Season	Water Temperature (°C)	Salinity	Nitrite (μg/L)	Nitrate (μg/L)	Ammonium (μg/L)	Phosphorus (μg/L)	Chl*a* (μg/L)
Spring	20.10 ± 1.11	29.572 ± 0.162	4.80 ± 1.99	76.46 ± 31.43	16.10 ± 4.68	1.76 ± 0.93	1.77 ± 0.74
Summer	22.34 ± 0.59	29.580 ± 0.162	4.57 ± 1.45	66.21 ± 33.83	9.86 ± 4.68	5.40 ± 2.65	3.74 ± 1.89
Autumn	14.21 ± 2.14	29.081 ± 0.441	25.06 ± 9.30	206.17 ± 78.66	26.63 ± 11.03	8.58 ± 6.40	1.26 ± 0.19
Winter	5.49 ± 0.75	29.733 ± 0.150	25.39 ± 8.55	182.92 ± 39.78	22.46 ± 5.47	4.17 ± 2.46	3.18 ± 0.65

**Table 2:** Taxa composition of zooplankton across seasons in this study.

Group	Spring	Summer	Autumn	Winter	Totally
Hydromedusa	8	9	4	1	10
Siphonophora	/	/	2	1	2
Ctenophora	/	/	1	/	1
Copepoda	8	5	8	5	12
Amphipoda	3	1	1	/	3
Sergestidae	/	2	/	/	2
Chaetognatha	1	1	1	1	1
Tunicata	1	1	2	/	2
Planktonic larvae	11	12	6	3	16
Totally	32	31	25	11	49

**Table 3:** Dominant zooplankton taxa and their dominance (*Y*) and abundance (*A*) values across seasons.

Scientific Name	Spring		Summer		Autumn		Winter	
	*Y*	*A*/(ind·m^−3^)	*Y*	*A*/(ind·m^−3^)	*Y*	*A*/(ind·m^−3^)	*Y*	*A*/(ind·m^−3^)
*Clytia hemisphaerica*	0.02	6.4	0.07	4.3	/	/	/	/
*Diphyes chamissonis*	/	/	/	/	0.08	4.9	/	/
*Muggiaea atlantica*	/	/	/	/	0.15	7.3	/	/
*Calanus sinicus*	0.07	89.4	/	/	/	/	0.14	3.0
*Paracalanus parvus*	/	/	/	/	0.08	8.4	/	/
Gammaridea	0.02	9.4	/	/	/	/	/	/
*Dolioletta gegenbauri*	/	/	/	/	0.08	7.2	/	/
*Aidanosagitta crassa*	0.18	28.8	0.02	2.3	0.32	23.3	0.71	10.2
Ophiopluteus larva	/	/	0.03	2.8	0.03	8.1	/	/
Polychaeta larva	/	/	0.05	2.8	0.05	4.7	/	/
Brachyura Zoea larva	0.03	6.7	0.05	3.3	/	/	/	/
Porcellana Zoea larva	/	/	0.16	12.2	/	/	/	/
Macrura larva	0.05	33.9	0.09	4.7	/	/	/	/
Fish egg	0.25	38.5	/	/	/	/	/	/
Fish larva	0.08	16.5	/	/	/	/	/	/

## Data Availability

Relevant information has been added in the article.

## Supplementary Materials

The following supporting information can be downloaded at: https://www.mdpi.com/article/10.3390/biology15090679/s1, Table S1: Depth (m) of the sampling stations in this study; Table S2: Environmental factor values in the coastal waters surrounding Lanshan Port; Table S3: List of zooplankton taxa from the coastal waters surrounding Lanshan Port.
