# Supplementary material for: Seasonal Variation in Zooplankton Community Structure and Its Environmental Drivers in the Coastal Waters of Lanshan Port
Source: Biology (Basel). 2026 Apr 25;15(9):679. doi: 10.3390/biology15090679 (PMC13162687; doi:10.3390/biology15090679)
Supplement: Supplementary file 1 [file biology-15-00679-s001.zip › Table S1.pdf]

Table S1 Depth (m) of the sampling stations in this study.

Stations	Spring	Summer	Autumn	Winter	Mean value
RL1	6.5	6.5	6.0	5.0	6.0
RL2	9.0	9.5	8.5	8.0	8.8
RL3	6.0	8.0	7.5	7.0	7.1
RL4	8.5	9.5	6.5	8.0	8.1
RL5	13.5	14.5	12.0	12.0	13.0
RL6	4.5	6.0	7.0	8.5	6.5
RL7	22.0	24.0	19.0	21.0	21.5
RL8	17.0	9.5	9.5	18.5	13.6
RL9	2.5	3.0	3.0	3.0	2.9
RL10	8.5	16.0	9.5	9.5	10.9
RL11	12.0	9.5	9.5	13.5	11.1
RL12	14.0	18.0	14.0	15.0	15.3
