# Supplementary material for: Seasonal Variation in Zooplankton Community Structure and Its Environmental Drivers in the Coastal Waters of Lanshan Port
Source: Biology (Basel). 2026 Apr 25;15(9):679. doi: 10.3390/biology15090679 (PMC13162687; doi:10.3390/biology15090679)
Supplement: Supplementary file 1 [file biology-15-00679-s001.zip › Table S2.pdf]

Table S2 Environmental factor values in the coastal waters surrounding Lanshan Port

Season	Environmental Factor	Sampling Station											
		RL1	RL2	RL3	RL4	RL5	RL6	RL7	RL8	RL9	RL10	RL11	RL12
Spring	Water Temperature (°C)	19.08	19.05	19.30	19.10	19.05	19.23	20.55	21.03	21.34	21.82	21.60	20.08
	Salinity	29.662	29.666	29.607	29.684	29.804	29.616	29.586	29.662	29.379	29.311	29.279	29.607
	Nitrite (μg/L)	4.56	3.30	10.7	4.44	3.18	4.87	3.78	3.60	6.43	4.68	4.87	3.24
	Nitrate (μg/L)	98.2	90.0	114	113	40.0	109	82.3	39.6	50.5	51.7	25.2	104
	Ammonium (μg/L)	21.6	12.7	24.3	11.9	13.0	13.1	12.4	17.1	19.2	20.1	8.1	19.7
	Phosphorus (μg/L)	0.908	0.908	1.19	1.48	0.624	1.76	1.76	2.89	1.48	3.46	1.48	3.18
	Chl *a* (μg/L)	2.42	1.41	2.98	1.01	1.19	1.08	1.41	1.47	3.1	1.86	2.19	1.07
Summer	Water Temperature (°C)	23.52	23.65	23.7	23.62	22.14	22.86	22.42	23.98	23.33	23.98	23.69	23.2
	Salinity	29.574	29.692	29.732	29.714	29.750	29.622	29.644	29.570	29.363	29.347	29.280	29.670
	Nitrite (μg/L)	1.47	4.72	5.73	4.52	5.06	7.83	3.84	3.97	4.18	5.67	4.11	3.70
	Nitrate (μg/L)	23	16.5	108	32.3	98.6	120	96.6	76.3	78.4	51.1	32.6	61.1
	Ammonium (μg/L)	12.2	1.19	7.49	8.10	7.1	19.5	16.6	7.93	8.66	11.5	12.1	5.98
	Phosphorus (μg/L)	9.34	7.21	7.21	7.49	8.06	1.53	2.95	5.90	1.36	3.91	6.18	3.63
	Chl *a* (μg/L)	8.10	2.25	2.20	2.52	3.15	5.54	3.77	2.65	6.23	3.36	2.42	2.64
Autumn	Water Temperature (°C)	12.45	12.27	12.38	12.35	12.30	12.54	13.18	16.45	16.72	16.90	16.70	16.30
	Salinity	29.168	29.335	29.268	29.321	29.416	29.287	29.321	29.141	27.827	29.123	28.654	29.114
	Nitrite (μg/L)	24.5	24.8	25.0	35.1	37.1	32.8	38.1	13.9	22.9	11.2	9.57	25.8
	Nitrate (μg/L)	324	180	233	242	176	253	124	156	379	130	154	123
	Ammonium (μg/L)	46.7	11.4	20.7	21.6	20.2	17.8	22.5	32.5	50.0	20.4	26.3	29.4

	Phosphorus (μg/L)	19.3	6.27	5.85	5.42	4.01	9.38	5.42	2.66	23.3	9.17	8.89	3.23
	Chl *a* (μg/L)	1.02	1.19	1.53	1.14	1.20	1.36	1.08	1.01	1.46	1.36	1.53	1.23
Winter	Water Temperature (°C)	3.96	5.28	4.28	5.62	6.32	5.74	5.86	6.24	5.16	6.38	5.52	5.46
	Salinity	29.444	29.723	29.722	29.597	29.936	29.763	29.845	29.791	29.769	29.560	29.683	29.964
	Nitrite (μg/L)	23.7	27.4	24	34.3	36.6	31.4	37.2	18.2	23.8	11.3	10.1	26.7
	Nitrate (μg/L)	250	168	204	196	151	215	135	148	259	166	156	147
	Ammonium (μg/L)	21.5	23.5	26.9	29.7	29.1	27.3	19.6	21.3	26.8	16.2	14.1	13.5
	Phosphorus (μg/L)	2.67	3.24	1.82	3.24	1.82	2.1	4.66	3.53	6.94	3.81	6.09	10.1
	Chl *a* (μg/L)	2.84	4.10	4.32	3.87	2.79	3.47	3.35	2.50	2.96	2.21	2.90	2.85
