# Supplementary material for: Seasonal Variation in Zooplankton Community Structure and Its Environmental Drivers in the Coastal Waters of Lanshan Port
Source: Biology (Basel). 2026 Apr 25;15(9):679. doi: 10.3390/biology15090679 (PMC13162687; doi:10.3390/biology15090679)
Supplement: Supplementary file 1 [file biology-15-00679-s001.zip › Table S3.pdf]

Table S3 List of zooplankton taxa from the coastal waters surrounding Lanshan Port in this study

No.	Scientific name	Taxon/Group	Spring	Summer	Autumn	Winter
1	*Clytia hemisphaerica*	Hydromedusa	+	+	+	+
2	*Turritopsis nutricula*	Hydromedusa	+	+		
3	*Zanclea apicata*	Hydromedusa	+	+		
4	*Eucheilota menoni*	Hydromedusa	+	+	+	
5	*Zanclea costata*	Hydromedusa	+			
6	*Bougainvillia muscus*	Hydromedusa	+	+		
7	*Sugiura chengshanense*	Hydromedusa	+	+	+	
8	*Muggiaea atlantica*	Siphonophora			+	+
9	*Eirene ceylonensis*	Hydromedusa		+		
10	*Diphyes chamissonis*	Siphonophora			+	
11	*Proboscidactyla flavicirrata*	Hydromedusa		+		
12	*Obelia* spp.	Hydromedusa	+	+	+	
13	*Pleurobrachia globosa*	Ctenophora			+	
14	*Calanus sinicus*	Copepoda	+		+	+
15	*Centropages abdominalis*	Copepoda	+		+	+
16	*Corycaeus* (*Ditrichocorycaeus*) *affinis*	Copepoda	+		+	+
17	*Centropages dorsispinatus*	Copepoda			+	
18	*Oithona similis*	Copepoda				+
19	*Acartia pacifica*	Copepoda	+	+	+	
20	*Eurytemora pacifica*	Copepoda	+			
21	*Calanopia thompsoni*	Copepoda		+	+	
22	*Labidocera rotunda*	Copepoda	+			
23	*Acartia omori*	Copepoda		+	+	
24	*Centropages tenuiremis*	Copepoda	+	+		
25	*Paracalanus parvus*	Copepoda	+	+	+	+
26	Gammaridea	Amphipoda	+	+	+	
27	*Acetes japonicus*	Sergestidae		+		
28	*Lucifer* spp.	Sergestidae		+		
29	Caprellidae	Amphipoda	+			
30	*Themisto gracilipes*	Amphipoda	+			
31	*Aidanosagitta crassa*	Chaetognatha	+	+	+	+
32	*Oikopleura dioica*	Tunicata	+	+	+	
33	*Dolioletta gegenbauri*	Tunicata			+	
34	Brachyura Megalopa larva	Planktonic larva		+		
35	Brachyura Zoea larva	Planktonic larva	+	+		
36	Polychaeta larva	Planktonic larva	+	+	+	+
37	Porcellana Zoea larva	Planktonic larva	+	+		
38	Gastropoda larva	Planktonic larva	+	+		
39	Bipinnaria larva	Planktonic larva			+	+
40	Cirripedia larva	Planktonic larva	+			
41	Copepodite Nauplius larva	Planktonic larva				+
42	Ophiopluteus larva	Planktonic larva	+	+	+	

43	Squilla Alima larva	Planktonic larva	+	+	
44	Bivalvia larva	Planktonic larva	+	+	+
45	Cephalopoda larva	Planktonic larva		+	
46	Macrura larva	Planktonic larva	+	+	+
47	Phoronis larva	Planktonic larva			+
48	Fish egg	Planktonic larva	+	+	
49	Fish larva	Planktonic larva	+	+	

Notes: Blanks indicate non-occurrence; “+” indicates occurrence.
